# Mumps: MMR vaccination and genetic diversity of mumps virus, 2007–2011 in Catalonia, Spain

**DOI:** 10.1186/s12879-019-4496-z

**Published:** 2019-11-09

**Authors:** Irene Barrabeig, Andrés Antón, Núria Torner, Tomàs Pumarola, Josep Costa, Àngela Domínguez, the Working Group for the Study for Mumps in Catalonia, Josep Álvarez, César Arias, Irene Barrabeig, Neus Camps, Mónica Carol, Pere Godoy, Ana Martínez, Sofia Minguell, Ignasi Parrón, Ma Rosa Sala, Ariadna Rovira, Núria Torner, Cristina Rius

**Affiliations:** 1 Epidemiological Surveillance and Response to Public Health Emergencies Unit in Barcelona South, Agency of Public Health of Catalonia, Generalitat of Catalonia., Hospital Universitari de Bellvitge, Edifici antiga escola d’infermeria, 3a planta, Feixa Llarga, s/n, 08907 L’Hospitalet de Llobregat, Spain; 2 CIBER Epidemiología y Salud Pública, Institut of Health Carlos III, Madrid, Spain; 3 Virology Unit, Centre de Diagnòstic Biomèdic, Hospital Clínic, Barcelona, Spain; 4 Department of Medicine, University of Barcelona, Barcelona, Spain; 5 CIBER Enfermedades Hepáticas y Digestivas, Institute of Health Carlos III, Madrid, Spain

**Keywords:** Mumps virus, Genotypes, Molecular surveillance, MMR vaccine, Laboratory diagnosis

## Abstract

**Background:**

Mumps is a vaccine-preventable disease but outbreaks have been reported in persons vaccinated with two doses of MMR vaccine.

The objective was to describe the demographic features, vaccination effectiveness and genetic mumps virus diversity among laboratory-confirmed cases between 2007 and 2011 in Catalonia.

**Methods:**

Cases and outbreaks of mumps notified to the notifiable diseases system of Catalonia between 2007 and 2011 retrospectively registered were included. Public health care centres provided written immunization records to regional public health staff to determine the vaccination history.

Saliva and serum specimens were collected from suspected cases for laboratory-confirmation using real-time reverse-transcriptase PCR (**rtRT-PCR**) or serological testing.

Phylogenetic analysis of the complete SH gene (316 nucleotides) and complete coding HN protein (1749 nucleotides) sequences was made.

Categorical variables were compared using the Chi-square or Fisher’s tests and continuous variables using the Student test. Vaccination effectiveness by number of MMR doses was estimated using the screening method.

**Results:**

During the study period, 581 confirmed cases of mumps were notified (incidence rate 1.6 cases/100,000 persons-year), of which 60% were male. Three hundred sixty-four laboratory-confirmed cases were reported, of which 44% were confirmed by **rtRT-PCR**. Of the 289 laboratory-confirmed cases belonging to vaccination cohorts, 33.5% (97) had received one dose of MMR vaccine and 50% (145) two doses.

Based on phylogenetic analyses of 316-nucleotide and 174-nucleotide SH sequences, the viruses belonging to viral genotypes were: genotype G (126), genotype D (23), genotype H (2), genotype F (2), genotype J (1), while one remained uncharacterized.

Amino acid differences were detected between circulating strains and the Jeryl Lynn vaccine strains, although the majority of amino acid substitutions were genotype-specific.

Fifty-one outbreaks were notified that included 324 confirmed mumps cases. Genotype G was the most frequent genotype detected. The family (35%), secondary schools (25%) and community outbreaks (18%) were the most frequent settings.

**Conclusions:**

Our study shows that genotype G viruses are the most prevalent in Catalonia. Most cases occurred in people who had received two doses of MMR, suggesting inadequate effectiveness of the Jeryl Lynn vaccine strain. The possible factors related are discussed.

## Background

Mumps is a highly-contagious vaccine-preventable disease caused by the mumps virus (MuV), an enveloped, negative-strand RNA virus belonging to the *Rubulavirus* genus of the *Paramyxoviridae* family. Infection is usually benign and self-limited, but is sub-clinical and asymptomatic in up to 30% of cases. The main clinical manifestation is parotitis, with one or both parotid glands involved. However, mumps infection may also result in clinical complications including aseptic meningitis, encephalitis and orchitis, among others. In countries with high vaccination coverages, mumps incidence has dropped dramatically as has the percentage of cases with encephalitis and other severe complications have been significantly reduced. Mumps disease shows epidemic peaks every 2 to 5 years [1]. Laboratory confirmation is based on the detection of MuV-specific immunoglobulin M (IgM) antibodies in serum or saliva specimens, by viral isolation in cell culture, or by detection of viral genomic RNA in clinical samples using molecular methods. Although MuV is considered to be serologically monotypic, distinct genetic lineages of wild-type MuV have been reported to be co-circulating. Up to 12 genotypes (A to N, excluding E and M) are currently recognised based on sequence analysis of the entire 316 nucleotides of the small hydrophobic (SH) gene, including the non-coding regions flanking the coding sequence of the SH protein [2]. The hemmagglutinin-neuraminidase (HN) gene encodes the protein that is the main target of neutralising antibodies. According to World Health Organization (WHO) guidelines, parallel analysis of the HN sequence further supports the assignment of genotyping based on SH gene sequencing, particularly when there is an ambiguous result [3, 4]. The WHO recommends MuV genotyping as a tool for the distribution of the genetic lineages that co-circulate worldwide and for viral epidemiological surveillance to trace the patterns of virus spread.

The combined measles, mumps and rubella vaccine (MMR) was included in the childhood immunization schedule at 12 months in 1980 in Catalonia, an autonomous community in the north-eastern Spain with 7.5 million inhabitants, and was covered by public financing. In 1987, administration of the first MMR dose was switched from 12 to 15 months, and reverted to 12 months in 2008. In 1988, a second dose of MMR was included at 11 years, which was switched to 4 years in 1998. The incidence of mumps decreased significantly from 456 to 3.6 cases per 100,000 persons-year between 1983 and 2011. Nevertheless, during the 2000s, several MuV outbreaks were reported in spite of the > 95 and > 90% vaccination coverages for the first and second doses, respectively, as reported in other countries with high vaccination coverages [5–9].

The objective of this study was to describe the demographic features, vaccination effectiveness and genetic MuV diversity of laboratory-confirmed cases from 2007 to 2011.

## Methods

### Patient population and study period

Descriptive study carried out with retrospectively registered cases. From January 2007 to December 2011, suspected cases of mumps were reported to the notifiable diseases system of Catalonia. WHO case definitions were used to define suspected and confirmed cases. A suspected case was defined as an acute onset of unilateral or bilateral tender, self-limiting swelling of the parotid or other salivary glands lasting ≥2 days without other apparent cause. A confirmed case was defined as a laboratory-confirmed case or a case meeting the clinical case definition and epidemiologically-linked to a confirmed case [10]. A mumps outbreak was defined as two or more cases linked by time and place within a maximum period of 26 days, one of which must be confirmed by a laboratory [11]. For each case (sporadic cases and cases related to outbreaks), field epidemiologists carried out an epidemiological survey, according to routine procedures for mumps surveillance in Catalonia, in which the following variables were collected: birth date, gender, symptom onset (parotitis), vaccination status, vaccine strain, sample collection date, and confirmatory diagnostic techniques. Public healthcare centres supplied regional public health staff with written immunization records to determine the vaccination history.

The vaccine administered in the study period was the Jeryl Lynn (JL) strain, except for a few years where the Rubini and Urabe AM9 strains were administered. We were unable to obtain the MMR vaccine composition used in each study patient, but the birth cohort of cases and the years when the strains were administered in Catalonia were used as an approximation. The Urabe strain was administered in 1991 and 1992. The first Rubini vaccine dose was administered between 1994 and 1995 (birth cohort 1993–1995), and the second Rubini vaccine dose in 1994 and 1996 (birth cohort 1983 to 1985). We did not consider the administration of one or two Rubini vaccine doses as valid immunization due to its low immunogenicity. Therefore, only cases vaccinated with the JL strain were included.

### Laboratory-confirmation

Serum and saliva specimens were collected from suspected cases for laboratory-confirmation using serological or real-time reverse-transcriptase PCR (**rtRT-PCR**) testing. Commercial enzyme-linked immunoassays (EIA) were used, according to the manufacturer’s recommendations, to detect MuV-specific IgG and IgM antibodies in serum specimens. A real-time one-step **RT-PCR** assay with primers and probe as previously described [12], was used to detect viral RNA in saliva specimens. A minimum of 1 ml was collected after stimulation of the area of the parotid and salivary glands for 30 s. Samples were collected preferably during the first 3 days of symptoms. rtRT-PCR was the test of choice in all patients, especially those previously-vaccinated, to avoid false-negative IgM antibody results in persons previously infected or immunized, regardless of the timing of sample [3, 13, 14]. Total nucleic acids were extracted from 200 μl of fresh specimen and eluted in 25 μl of RNase-free elution buffer using bioMérieux NucliSense easyMAG (bioMérieux, Marcy l’Etoile, France) according to the manufacturer’s instructions. Laboratory-confirmed specimens were kept frozen at − 80 °C for further analysis.

### Phylogenetic analysis

The complete SH gene with flanking non-coding regions (316 nucleotides) and complete coding HN protein (1749 nucleotides) sequences was sequenced in parallel for subsequent phylogenetic analyses to determine the MuV genotype of laboratory-confirmed viruses. Amplification of both viral regions was performed using the One-step RT-PCR Kit (Qiagen, Hilden, Germany) for one-step RT-PCR, the PCR Master Mix (Promega, Madison, USA) for nested-PCR, and the primers and PCR protocols as shown in Table 1. When there was non-amplification of the 316-nucleotide SH region, the complete coding SH sequence (174 nucleotides) was amplified using the nested-PCR protocol described by Palacios et al. [15]. PCR products were purified using Exo-SAP-IT (USB, Affymetrix Inc. Cleveland, Ohio, USA) and sequenced using the ABI Prism Big Dye Terminator cycle sequencing kit v3.1 on the ABI PRISM 3130XL sequencer (Applied Biosystems, Foster City, California, USA) using sequencing primers (Table 1) [15]. Nucleotide sequences were edited and assembled using SeqScape v2.5 software (Applied Biosystems, Foster City, California, USA) [12].

**Table 1:** Primer sequences and protocols for PCR amplification of complete coding SH gene with flanking non-coding sequence (316 nucleotides) and complete coding HN protein sequences. Nucleotide positions are relative to AF338106 (major component Jeryl-Lynn of live vaccine). The M13 primer binding sites used for sequencing are marked in bold

Primer Name	Primer Sequence (5′ - 3′)	Position (AF338106)
Amplification PCR Protocol		
Outer PCR reactions		
MuV_0Fa	CAAAACAAATCATATCAAYACAATATCAAG	6105–6134
MuV_0Fb	GGCTTAYATTGCRACYAAAGA	6066–6086
MuV_0Rab	TARGAGTATCTCATTTAGGCC	8464–8444
Thermal profile: 45 °C × 30 min - 95 °C × 15 min - 40 cycles (94 °C × 30 s - 50 °C × 1 min - 68 °C × 3 min) - 68 °C × 10 min		
Inner PCR reactions		
MuV_SH_1F	**TGTAAAACGACGGCCAGT**TCRAGTAGTGTCGATGATCTCAT	6130–6152
MuV_SH_1R	**CAGGAAACAGCTATGACC**TTRCTCAAGCCTTGRTCATT	6810–6791
MuV_HN_2F	**TGTAAAACGACGGCCAGT**TYCGRACCTGYTTCCGAATA	6699–6718
MuV_HN_2R	**CAGGAAACAGCTATGACC**ACTGTTGCAATYGAGCAG	7359–7342
MuV_HN_3F	**TGTAAAACGACGGCCAGT**CATAATGTRATTAATGCCAACTG	7196–7218
MuV_HN_3R	**CAGGAAACAGCTATGACC**CACCAGCTRGTACTYCTCTG	7893–7874
MuV_HN_4F	**TGTAAAACGACGGCCAGT**TGYATTYCTDGTCTGTGCYTG	7744–7764
MuV_HN_4R	**CAGGAAACAGCTATGACC**GCCATTCTGGCCTGTT	8442–8427
Thermal profile: 95 °C × 5 min - 35 cycles (95 °C × 30 s - 50 °C × 30 s - 72 °C × 1 min) - 72 °C × 10 min		
Sequencing protocol		
M13F	TGTAAAACGACGGCCAGT	
M13R	CAGGAAACAGCTATGACC	
Thermal profile: 96 °C × 1 min - 30 cycles (96 °C × 10 s - 50 °C × 5 s - 60 °C × 4 min)		

Phylogenetic analyses of SH and HN sequences were carried out using the reference genotype sequences according to WHO genotyping guidelines for mumps [2]. Molecular evolutionary models of nucleotide substitution were fitted to the multiple sequence alignments using the evolutionary analyses conducted in MEGA v5.2 [16]. The phylogenetic trees were reconstructed using a neighbour-joining distance method as implemented in MEGA v5.2 [16], with the evolutionary model with the lowest Bayesian information criterion score. The topological accuracy of the trees was evaluated by the bootstrap method (1000 replicates).

The amino acid composition of the complete HN protein sequence was characterised relative to the homologous sequences of vaccine strains with accession numbers AF338106 (major JL component of live vaccine), AF345290 (minor JL component of live vaccine) and X93180 (Rubini vaccine strain) using MEGA v5.2 [16]. Nucleotide sequences were collapsed to haplotypes using ALignment Transformation EnviRonment (ALTER) to reduce redundant information [17] before being translated to amino acid sequences to reveal the different circulating genetic MuV variants even though translated amino acid sequences were similar. The acquisition or loss of potential N-glycosylation sites in the complete amino acid sequence of the HN protein was tracked using N-GlycoSite (www.hiv.lanl.gov). In addition, the genetic variability of SH and HN sequences was studied within genotypes, between genotypes and relative to the major JL component of the live vaccine (AF338106).

### Statistical analysis

Categorical variables were compared using the Chi-square or Fisher’s tests and continuous variables using the Student test. Values of *p* < 0.05 were considered to be statistically significant.

Vaccination effectiveness (VE) was calculated using all confirmed cases born between 1982 and 2010. We excluded (a) children aged < 12 or 15 months according to the current childhood immunization schedule, (b) people with unknown vaccination status and (c) people who had received mumps-containing vaccine within 14 days of the onset of mumps symptoms. VE was estimated using 433 eligible cases out of 581 confirmed cases. According to the methodology described by Orenstein et al. [18], VE was estimated using the screening method by the following formula: VE_i_ = 1- ((PCV_i_/1-PCV_i_) x (1-PPV_i_)/PPV_i_)), where PCV_i_ is the proportion of cases with *i* doses, PPV_i_ is the proportion of the population vaccinated with *i* doses, and *i* is 1 or 2. 95% confidence intervals (CI) were calculated using the Taylor series. To estimate the VE of one dose, people who had received two doses were excluded from the calculations of the proportions of cases and the population vaccinated. Similarly, people who had received one dose were excluded from calculations that estimated the effectiveness of two doses.

The statistical analysis was made using SPSS/PC, version 18.0 for Windows (SPSS Inc., Chicago, IL, USA) and Epidat.

## Results

### Case notifications

From January 2007 to December 2011, 1175 suspected cases of mumps disease were reported to the notifiable diseases system of Catalonia, of which 581 (49%) were confirmed cases (incidence rate of 1.6 cases/100,000 persons-year) (Table 2). 60% (347) were male with a mean age 16.7 years (SD ± 10.9 years) and 40% (234) were female with a mean age of 18.2 years (SD ± 13.6 years) (*p* = 0.08).

**Table 2:** Characteristics of confirmed mumps cases and outbreaks according to circulating virus genotypes. Catalonia 2007–2011

CASES	Confirmed cases		Laboratory-confirmed cases		Genotype G		Genotype D		Others^a^	
	N	*%*	N	*%*	N	*%*	N	*%*	N	*%*
*Gender*	581		364		126		23		5	
Male	347	*59.7%*	201	55.2%	78	*61.9%*	11	*47.8%*	4	*80.0%*
Female	234	*40.3%*	163	44.8%	48	*38.1%*	12	*52.2%*	1	*20.0%*
*Age group (years)*										
< 1	4	*0.7%*	2	*0.5%*	0	*0.0%*	0	*0.0%*	0	*0.0%*
1–4	69	*11.9%*	34	*9.3%*	3	*2.4%*	5	*21.7%*	0	*0.0%*
5–14	209	*36.0%*	116	*31.9%*	32	*25.4%*	13	*56.5%*	3	*60.0%*
15–24	173	*29.8%*	111	*30.5%*	56	*44.4%*	1	*4.3%*	0	*0.0%*
25–34	75	*12.9%*	78	*21.4%*	28	*22.2%*	4	*17.4%*	1	*20.0%*
≥ 35	51	*8.8%*	23	*6.3%*	7	*5.6%*	0	*0.0%*	1	*20.0%*
*Year*										
2007	272	*46.8%*	204	*56.0%*	90	*71.4%*	1	*4.3%*	1	*20.0%*
2008	85	*14.6%*	56	*15.4%*	2	*1.6%*	10	*43.5%*	3	*60.0%*
2009	56	*9.6%*	22	*6.0%*	8	*6.3%*	1	*4.3%*	1	*20.0%*
2010	68	*11.7%*	30	*8.2%*	5	*4.0%*	8	*34.8%*	0	*0.0%*
2011	100	*17.2%*	52	*14.3%*	21	*16.7%*	3	*13.0%*	0	*0.0%*
*Laboratory tests*	364	*62.6%*								
**rtRT-PCR** assays			160	*44.0%*	108	*85.7%*	22	*95.7%*	4	*80.0%*
IgM assays			166	*45.6%*	–		–		–	
Both tests			30	*8.2%*	18	*14.3%*	1	*4.3%*	1	*20.0%*
Seroconversion			8	*2.2%*	–		–		–	
Epidemiologically-linked cases	217	*37.4%*	364							
*Vaccination status*										
Non-vacinated	150	*25.8%*	110	*30.2%*	34	*27.0%*	8	*34.8%*	2	*40.0%*
One MMR dose	85	*14.6%*	97	*26.6%*	39	*31.0%*	4	*17.4%*	0	*0.0%*
Two MMR doses	314	*54.0%*	145	*39.8%*	52	*41.3%*	10	*43.5%*	3	*60.0%*
Unknown number	15	*2.6%*	4	*1.1%*	1	*0.8%*	0	*0.0%*	0	*0.0%*
Missing information	17	*2.9%*	8	*2.2%*	0	*0.0%*	1	*4.3%*	0	*0.0%*
*Complications*										
Orchitis	20	*3.4%*	19	*5.2%*	7	*5.6%*	0	*0.0%*	0	*0.0%*
Encephalitis	3	*0.5%*	3	*0.8%*	0	*0.0%*	0	*0.0%*	0	*0.0%*
Fever	344	*59.2%*	235	*64.6%*	83	*65.4%*	7	*30.4%*	3	*100.0%*
*Time since second dose of MMR vaccine*										
0–5 years	89	*28.3%*	45	*31.1%*	9	*17.3%*	4	*40.0%*	1	*33.3%*
≥ 6 years	225	*71.7%*	100	*68.9%*	43	*82.7%*	6	*60.0%*	2	*66.7%*
OUTBREAKS	Number of outbreaks		Outbreak related cases		Genotype G		Genotype D		Others^a^	
	N	*%*	N	*%*	N	*%*	N	*%*	N	*%*
Total outbreaks	51^b^	*_*	*324*	*_*	20	*_*	4	*_*	3	*_*
*Year*										
2007	31	*60.8%*	166	*51.2%*	16	*80.0%*	0	*0.0%*	1	*33.3%*
2008	11	*21.6%*	39	*12.0%*	0	*0.0%*	2	*50.0%*	2	*66.7%*
2009	2	*3.9%*	9	*2.8%*	2	*10.0%*	0	*0.0%*	0	*0.0%*
2010	3	*5.9%*	19	*5.9%*	1	*5.0%*	2	*50.0%*	0	*0.0%*
2011	4	*7.8%*	91	*28.1%*	1	*5.0%*	0	*0.0%*	0	*0.0%*
*Setting*										
Family	18	*35.3%*	42	*13.0%*	7	*35.0%*	1	*25.0%*	0	*0.0%*
Secondary	13	*25.5%*	56	*17.3%*	3	*15.0%*	1	*25.0%*	1	*33.3%*
High school	8	*15.7%*	168	*51.9%*	3	*15.0%*	2	*50.0%*	1	*33.3%*
Occupational	3	*5.9%*	7	*2.2%*	1	*5.0%*	0	*0.0%*	1	*33.3%*
Community (discotheque, football team, neighbours)	9	*17.6%*	51	*15.7%*	6	*30.0%*	0	*0.0%*	0	*0.0%*

^a^Others: Genotype F, Genotype H and Genotype J

^b^In 21 outbreaks the genotype was not known and 3 outbreaks were not typable”

Fifty-nine percent of confirmed cases had a temperature (≥38 °C). 3% presented orchitis and 0.5% encephalitis as complications and 24 patients were hospitalized (median 2 days range: 1–5 days) (Additional file 1: Table S1).

Of the 364 laboratory-confirmed cases, 160 (44%) were confirmed by **rtRT-PCR** assay, 174 (48%) by serological assays and 30 (8%) using both techniques (Table 2). 55% (201) of laboratory-confirmed cases were male and the mean age was 19.3 years, SD ± 12.6 years (20.2 years in females **vs. 18.6** years in males, *p* = 0.2) and 217 were epidemiologically linked to a laboratory-confirmed case.

### Vaccination status and vaccine effectiveness

Of the 364 laboratory-confirmed cases, 297 (81.6%) had criteria for MMR vaccination as they were born after 1980, when the childhood immunization schedule was introduced in Catalonia. Of these, 49% (145) were vaccinated with two doses, 32.5% (97) with one dose, 14.5% (43) did not receive MMR, the number of doses was unknown in 1.3% (4) and information was missing in 2.7% (8). Of the 67 remaining cases, 64 were born before 1980 and had had no opportunity for vaccination and three cases occurred in children aged < 1 year (Table 2).

The VE was estimated in the 433 eligible cases out of the 581 confirmed cases reported in Catalonia during 2007–2011. The point estimate of VE for one dose ranged between 86.2% and 87.1% and for two doses between 87.6% and 89.3% (Table 3).

**Table 3:** Estimates of vaccine effectiveness for one and two doses of the MMR vaccine by birth cohort of all confirmed cases of mumps. Catalonia 2007–2011

	Number of cases			Vaccine effectiveness (95%CI)	
Birth cohort	No vaccine	One dose	Two doses	One dose	Two doses
1982–1991	32	35	73	87.1 (79.2–92,1)	89.3 (83.7–92.9)
1992–2001^a^	10	52	132	86.8 (74.1–93.3)	87.6 (75,4- 93,7)
2002–2010^b^	12	51	36	86.2 (74.2–92.7)	88.5 (76.1–94,4)

*n* = 581 (433 cases targeted for vaccination; 116 cases non-targeted for vaccination, 15 unknown number and 17 cases missing information)

^a^ To calculate the effectiveness of the second dose, birth cohorts from 1994 to 1996 were excluded as the MMR vaccine administered contained the Rubini strain

^b^ Estimates of VE for two doses were birth cohorts from 2002 to 2008

### Distribution of MuV genotype

**Fig. 1:**
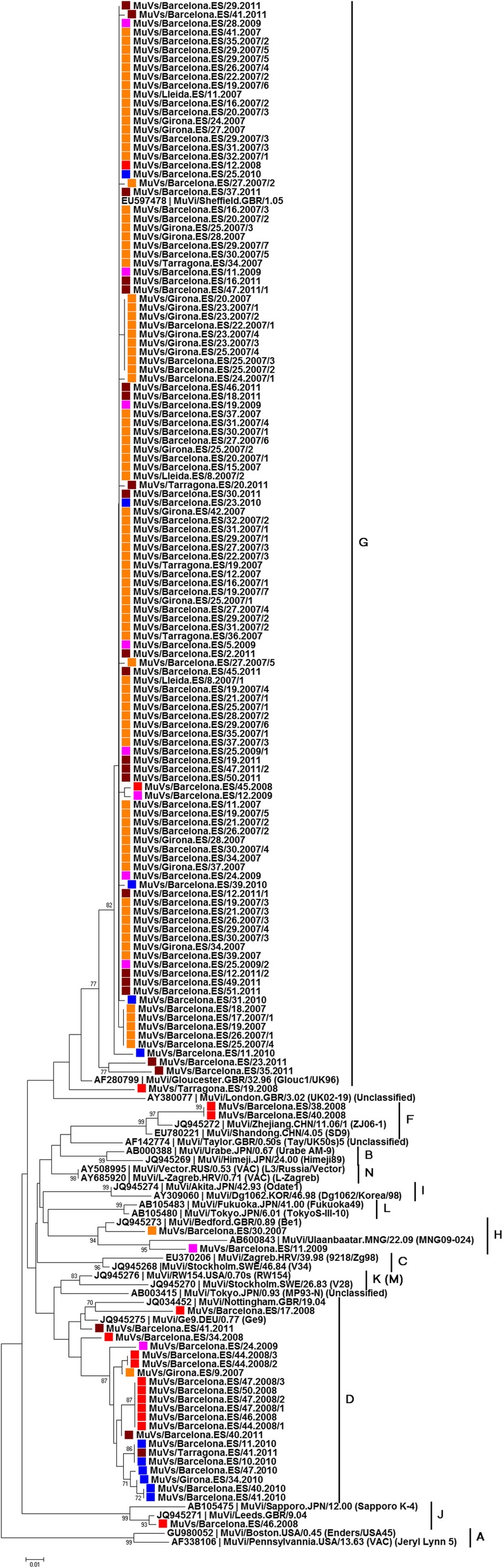
Phylogenetic reconstruction of complete coding SH gene. The strains in the present study are coloured by year: 2007 (orange), 2008 (red), 2009 (pink), 2010 (blue) and 2011 (brown)

According to WHO guidelines [2] MuV genotyping was successful in 147 (77%) of the 190 cases confirmed by **rtRT-PCR** by phylogenetic analysis of complete coding SH protein sequences with flanking non-coding regions (316 nucleotides) (Fig. 1). In addition, phylogenetic analysis was also performed in another 8 (4%) cases based on complete coding SH sequences (174 nucleotides) (Additional file 4: Figure S1), showing robust, phylogenetic analysis results (bootstrap values > 70%) even though the length of sequences was shorter than recommended by the WHO for characterisation. According to the phylogenetic analyses of the 316-nucleotide (Fig. 1) or 174-nucleotide (Additional file 4: Figure S1) SH sequences, genotype frequencies were: 126 (81%) genotype G, 23 (15%) genotype D, 2 (1%) genotype H, 2 (1%) genotype F, 1 (< 1%) genotype J and 1 (< 1%) unclassified (Table 2). MuV genotyping based on complete coding HN sequences (Additional file 5: Figure S2) was consistent with the results obtained from SH sequences. The remaining 35 (18%) cases of MuV could not be genotyped due to non-amplification of SH or HN regions, likely due to the low viral load or the low quality of genetic material from laboratory-confirmed clinical samples.

The genetic variability of nucleotide and deduced amino acid SH and HN sequences of laboratory-confirmed samples was also studied (Additional file 2: Table S2). The 155 complete 174-nucleotide SH sequences were collapsed into 35 (23%) haplotypes, while the 110 complete coding HN sequences were collapsed into 38 (35%) haplotypes despite being longer (1749 nucleotides). Within genotypes, the complete coding SH sequences showed greater mean genetic divergences (0.49–6.90%) than the complete coding HN sequences (0.18–3.09%). The mean genetic distances between sequences within genotypes relative to the sequences from the major component of the JL vaccine strain are shown as additional information (Additional file 2: Table S2).

The deduced amino acid sequences of the complete coding HN region that were collapsed into haplotypes were compared with the major component of the JL and other vaccine strains (Table 4). Up to 6 amino acid differences were found, but there was no gain or loss of potential N-glycosylation positions within antigenic regions (amino acid positions 265–288, 329–340 and 352–360) previously characterised. In addition, up to 9 other amino acid changes were found within other viral HN regions where some amino acid substitutions were previously related to immune escape from neutralisation [19]. No amino acid changes (K335E/R, P/Q354H, E/D356S, R360C, N464K, and S466 N) in other sites previously associated with neurovirulence were detected [20].

**Table 4:**
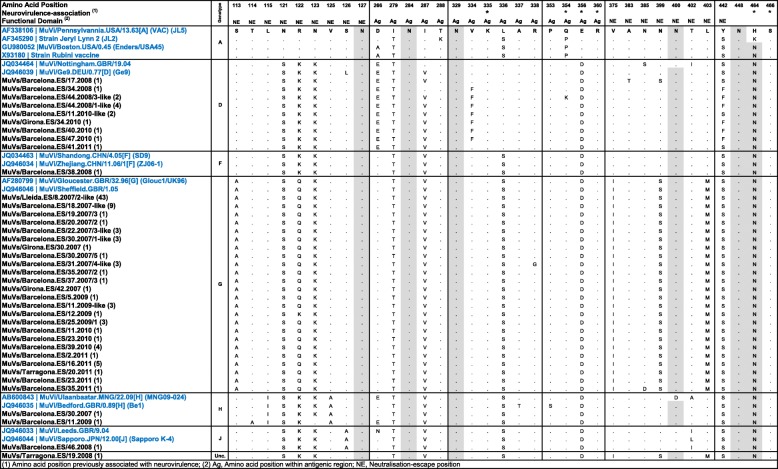
Molecular characterization of deduced amino acid positions in HN protein. The nucleotide sequences were previously collapsed into haplotypes, and the numbers of sequences represented are shown in brackets with the name in the first column. Potential n-glycosylation positions are highlighted in grey. Amino acids other than the deduced reference sequence (AF338106) are shown with a dot

Amino acid position previously associated with neurovirulence; *Ag* Amino acid position within antigenic region, *NE* Neutralisation-escape position

Nucleotide sequences were submitted to the GenBank database (accession numbers KX609797-KX609951).

### Temporal distribution of outbreaks

In 2007–2011, 51 outbreaks of mumps were notified, with 324 confirmed cases. Most occurred in 2007 (61%) and 2008 (22%). The most frequent settings were the family (35%), with a mean size of 2.3 cases, secondary schools (25%) with a mean size of 4.3 cases, and community outbreaks in young adults (discotheque, football team, etc.) (18%) with a mean size of 5.7. Eight (16%) outbreaks occurred in high schools, with a mean size of 21 (Table 2).

**Fig. 2:**
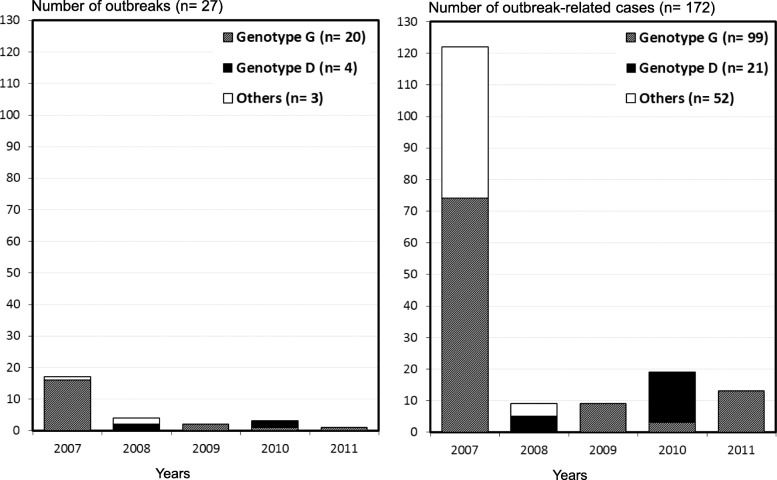
Distribution of outbreaks of mumps and outbreak-related cases according to genotype and year of detection. Catalonia 2007–2011. Others: Genotype F, Genotype H and Genotype J

Phylogenetic analysis was carried out in 30 outbreaks: the genotype detected was G in 20 outbreaks, D in 4 outbreaks and F, H and J in one outbreak each, while three outbreaks were non-typable (Table 2 and Fig. 2).

Genotype G viruses circulated throughout the study period at variable levels, affecting 99 confirmed cases (Fig. 2). In 2007, genotype G viruses were predominant with 16 (80%) outbreaks that affected 74 confirmed cases, constituting an epidemic wave but during 2008–2011 their circulation decreased (3 outbreaks with 25 confirmed cases). This was considered an inter-epidemic period. Genotypes D, F, H and J also co-circulated during the inter-epidemic period (Fig. 2).

## Discussion

This study found five different MuV genotypes. Of the more than 1100 suspected cases, almost half were confirmed by laboratory methods or epidemiological linkage. In clinical parotitis, causes other than the mumps virus should be considered. In a previous study, we found that approximately 51% of suspected cases of mumps infection were laboratory-confirmed for infection by other viruses such as the Epstein-Bar virus, parainfluenza viruses or adenovirus [21].

About 70% of laboratory-confirmed mumps cases were vaccinated, with a mean age of 14.3 years (SD ± 7.3), of whom 40% received two recommended JL vaccine doses. Phylogenetic analyses of SH and HN sequences revealed that the genotypes most frequently detected were G and D. In Spain, the emergence and spread of genotype G viruses since 2006 has been reported [22], and virological surveillance shows continuity until 2011 due to the findings of the present study. The circulation of the genotype G virus has been reported in outbreaks in Europe and USA, and particularly in patients vaccinated with two doses of MMR [6, 7, 19, 23–27]. Our results suggest that genotype G viruses are highly prevalent and have a substantial capacity to spread among the vaccinated population. Mumps cases have continued to occur after our study period, presenting as multiannual waves. Recent data suggest that in 2015, a new epidemic wave began in Catalonia, with genotype G being the most prevalent. This behaviour was also observed in the rest of Spain, where 57% of cases with a vaccination history had received ≥1 vaccine dose [28, 29].

A possible explanation might be the antigenic differences between the circulating and vaccine strains (immune escape) [30, 31], or progressive loss of protective antibodies over time (waning immunity). Other authors have suggested high-density, close-contact environments such as schools or universities as likely causes [6, 9, 32].

With respect to the differences detected between the circulating and JL vaccine strains, the alignment of deduced amino acid sequences revealed that most amino acid substitutions were genotype-specific.

Some reports suggest that neutralising antibodies are specific to the vaccine strain used, and humoral protection is not sufficiently efficient to prevent infection by MuVs from different genotypes and disease progression. Geographical differences between circulating genotypes and the vaccine strain have been reported worldwide. In the Western Hemisphere, the composition is mainly based on the JL strain (genotype A) and, in a few countries, on the Urabe AM9 (genotype B) and Leningrad-Zagreb (genotype N) strains, whereas the wild-type viruses currently circulating predominantly belong to genotype G strains. Some authors have **found** that neutralising epitopes were vaccine strain-specific and, therefore, vaccination did not completely prevent mumps disease and complications by viruses belonging to genotypes other than the vaccine genotypes [31]. Our results show that viruses belonging to five genotypes were co-circulating in the study period at varying levels despite high community vaccination coverage with the JL vaccine (mean of 94.2% for the second dose). Although our results suggest immune escape by the acquisition of amino acid substitutions within the antigenic epitopes of the HN protein, they also indicate a possible loss of immunity or secondary vaccine failure. Several factors support this explanation. First, waning immunity was linked to the time since vaccination [26, 33]. In our study, 72% of confirmed cases received the second dose of MMR ≥6 years before symptom onset. Secondly, the VE in observational studies of the JL strain (75%–82% for a single dose and 79%–95% for two doses) [3, 34–39] is lower than the efficacies reported in clinical trials (92%–96%) [40, 41]. Similarly, the point estimate of VE for all confirmed cases during the study period ranged between 86% and 87% for one dose and 88%–89% for two doses. Thirdly, 58% of laboratory-confirmed patients were aged > 15 years, and only 10% were aged < 5 years; this is consistent with other studies that found an upsurge of cases in young adults [24, 25], which is in accordance with a decrease in neutralising antibody levels over time, as reported in seroepidemiological studies, which probably result in incomplete protection against heterologous MuV strains [24, 42]. This may be explained, at the beginning of the vaccination period, by the fact that vaccinated persons had natural reinfection due to the circulating virus. When rates of disease incidence fell to low levels, the possibility of boosting was reduced. In Finland, a vaccination coverage of > 95% maintained over time permitted the elimination of endemic transmission of the virus in 1996 [43]. If low titres of neutralising antibodies are an important factor in outbreaks, it is essential to have a threshold titre to determine the response of anti-mumps antibodies and whether the subject is fully protected against wild virus infection [44].

Outbreaks have been reported in populations vaccinated with the JL vaccine strain, but also with the Urabe AM9 and Leningrad-Zagreb vaccines [45]. Therefore, the development of a new mumps vaccine would probably not be the solution to the current problem. Instead, revaccination with a third vaccine dose in adolescents could repair the loss of immunity, as other authors have reported [1]. At present, the CDCs recommend a third dose of MMR as a post-exposure measure to control outbreaks [33, 46–48].

We found that SH and HN sequencing provided the same genotyping results in most MuV infections. The comparison of mean genetic divergence within genotypes, the genetic distances relative to the JL vaccine strain, and the percentage of collapse into haplotypes, revealed that the coding SH protein sequence is more variable than the coding HN protein sequence at the nucleotide and amino acid levels, as reported by other authors [19]. Despite the lower diversity of the HN protein compared with the SH protein, its molecular characterisation is highly recommended to detect viral variants with changes that affect mainly antigenic epitopes [49].

The availability of nucleotide sequences might help to trace the person-to-person chain of transmission in epidemiological investigations of outbreaks in the future. A global sequence database and mumps strain bank similar to the Measles Nucleotide Surveillance (MeaNS) database should be developed to facilitate the distribution of sequence variants of MuV, which is particularly important to link endemic cases to imported cases from other countries and monitor the spread of novel genetic viral variants with new antigenic features on the HN or the F proteins.

One limitation of the study is that detection of the virus by **rtRT-PCR** was more frequent in persons who had received two doses of MMR than in unvaccinated persons. This might be due to the fact that, although **rtRT-PCR** is the test of choice, if epidemiologists know that the suspected case had received the vaccine they prioritize a **rtRT-PCR** test because, in vaccinated people, false-negative serology results (IgM antibody) are frequent (Additional file 3: Table S3).

## Conclusions

Our study, carried out with retrospective cases, shows that genotype G viruses are the most prevalent in Catalonia and may be transmitted within a highly-vaccinated population. Most cases occurred in people who had received two doses of MMR, suggesting inadequate effectiveness of the JL vaccine strain. The possible factors related to the decrease in vaccine effectiveness include secondary vaccine failure (waning immunity), intense exposure to the virus due to social overcrowding, and a possible mismatch between the vaccine genotype and that of circulating mumps virus strains.

Molecular and epidemiological studies are needed to provide information on the factors related to vaccine failure in countries with high vaccine coverages.

## Supplementary information

**Additional file 1: Table S1.** Rates of mumps complications and hospitalization according to MMR status. Catalonia 2007–2011. * Calculated in laboratory-confirmed cases.

**Additional file 2: Table S2.** Genetic divergences of complete coding SH and HN sequences of the present study at nucleotide and amino acid level. (1) The proportion (%) of nucleotide or amino acid differences per site from averaging over all sequence pairs within each group are shown.(2) Mean genetic distances (%) between genotypes and reference JL5 sequences (AF33106) are shown: n/c denotes cases in which it was not possible to estimate the evolutionary distance.

**Additional file 3: Table S3.** Characteristics of samples studied according to vaccination status.

**Additional file 4: Figure S1.** Phylogenetic reconstruction of complete coding SH protein (174 nucleotides, from 6268 to 6441positions in AF338106) using the neighbour-joining method rooted to strains belonging to genotype A. The strains of the present study are coloured by year: 2007 (orange), 2008 (red), 2009 (pink), 2010 (blue) and 2011 (brown).

**Additional file 5: Figure S2.** Phylogenetic reconstruction of complete coding HN protein sequences (1749 nucleotides, from 6614 to 8362 positions in AF338106) using the neighbour-joining method rooted to strains belonging to genotype A. The strains of the present study are coloured by year: 2007 (orange), 2008 (red), 2009 (pink), 2010 (blue) and 2011 (brown).

## Data Availability

The datasets used and analysed during the current study are available from the corresponding author on reasonable request.

## Abbreviations

Ag: Antigenic
CI: Confidence intervals
EIA: Enzyme-linked immunoassays
HN: Hemmagglutinin-neuraminidase
IgG: Immunoglobulin G antibodies
IgM: Immunoglobulin M antibodies
JL: Jeryl Lynn strain
MMR: Measles, mumps and rubella vaccine
MuV: Mumps virus
NE: Neutralization escape
OR: Odds ratio
PCR: Polymerase chain reaction
**rtRT-PCR**: **real time** reverse transcription polymerase chain reaccion
SH: Small hydrophobic
VE: Vaccine effectiveness
WHO: World Health Organization
