# Supplementary figures and images for: Mumps: MMR vaccination and genetic diversity of mumps virus, 2007–2011 in Catalonia, Spain
Source: BMC Infect Dis. 2019 Nov 9;19:954. doi: 10.1186/s12879-019-4496-z (PMC6842476; doi:10.1186/s12879-019-4496-z)

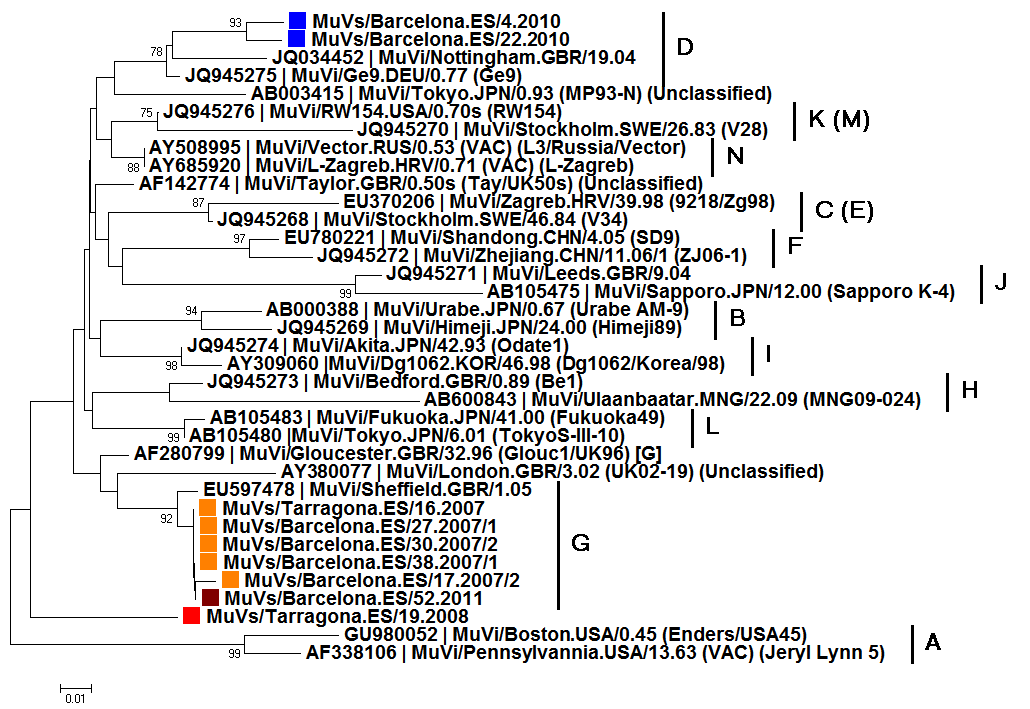

Supplement: Supplementary file 4 — Additional file 4: Figure S1. Phylogenetic reconstruction of complete coding SH protein (174 nucleotides, from 6268 to 6441positions in AF338106) using the neighbour-joining method rooted to strains belonging to genotype A. The strains of the present study are coloured by year: 2007 (orange), 2008 (red), 2009 (pink), 2010 (blue) and 2011 (brown). [file 12879_2019_4496_MOESM4_ESM.png]

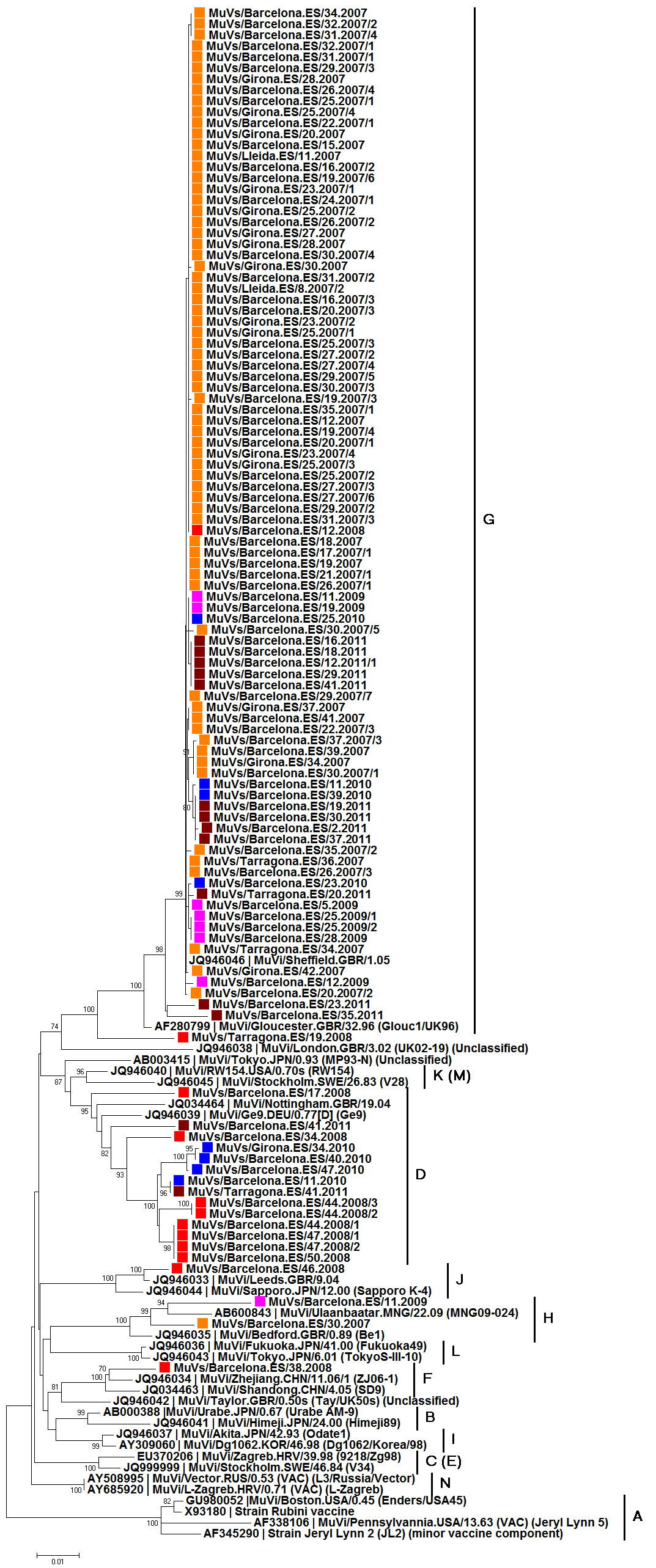

Supplement: Supplementary file 5 — Additional file 5: Figure S2. Phylogenetic reconstruction of complete coding HN protein sequences (1749 nucleotides, from 6614 to 8362 positions in AF338106) using the neighbour-joining method rooted to strains belonging to genotype A. The strains of the present study are coloured by year: 2007 (orange), 2008 (red), 2009 (pink), 2010 (blue) and 2011 (brown). [file 12879_2019_4496_MOESM5_ESM.png]
